# Clinical outcomes of FFR and IVUS-guided PCI in patients with myocardial bridging and proximal LAD stenosis

**DOI:** 10.3389/fcvm.2025.1648221

**Published:** 2026-01-12

**Authors:** Xi Wu, Mingxing Wu, Haobo Huang, Zhe Liu, He Huang, Lei Wang

**Affiliations:** Department of Cardiology, Xiangtan Central Hospital (the Affiliated Hospital of Hunan University), Xiangtan, Hunan, China

**Keywords:** myocardial bridging, fractional flow reserve, intravascular ultrasound, percutaneous coronary intervention, left anterior descending

## Abstract

**Background:**

Myocardial bridging (MB), once considered benign, is increasingly recognized for its role in myocardial ischemia, especially when coexisting with proximal left anterior descending (LAD) artery stenosis. Optimal revascularization strategies remain uncertain for such dual pathology. This study assessed whether a fractional flow reserve (FFR)-guided and intravascular ultrasound (IVUS)-optimized percutaneous coronary intervention (PCI) approach improves outcomes in this population.

**Methods:**

In this retrospective single-center study, 238 patients with moderate MB and proximal intermediate LAD stenosis were enrolled. Patients were stratified based on FFR measurements: those with FFR > 0.80 received medical therapy alone (*n* = 96), while patients with FFR ≤ 0.80 underwent IVUS-guided PCI (*n* = 142). Baseline characteristics, procedural data, and two-year follow-up outcomes were compared. Major adverse cardiovascular events (MACE) were recorded, and multivariate regression analysis identified predictors of poor outcomes.

**Results:**

Patients undergoing PCI (FFR ≤ 0.80) had significantly lower MACE rates than those managed conservatively (7.7% vs. 18.8%, *p* = 0.019), mainly due to reduced angina-related rehospitalization. PCI was an independent protective factor (Hazard Ratio = 0.526, *p* = 0.034). Among PCI patients, stent extension into the MB segment was linked with higher MACE incidence (18.6% vs. 3.0%, *p* = 0.001). IVUS revealed that stent extension correlated with severe MB compression, shorter distance between lesions, and more frequent dissections. Two anatomical factors—short MB-proximal lesion distance and MB dissection—were predictive of poor outcomes post-MB stenting.

**Conclusions:**

An FFR-guided, IVUS-supported PCI strategy improves clinical outcomes in patients with MB and proximal LAD stenosis, particularly when avoiding stent placement in dynamically compressed MB segments. Procedural planning using IVUS and careful lesion assessment is essential. Functional evaluation alone may underestimate ischemia in MB; integration of anatomical and diastolic functional indices is recommended.

## 1 Introduction

Although coronary angiography (CAG) has historically served as the gold standard for evaluating coronary artery stenosis, accumulating evidence highlights a frequent discordance between angiographic severity and the actual presence of functional ischemia (1). As a result, there is an increasing emphasis on integrating both anatomical and physiological data to improve the accuracy of revascularization decision-making (2). Fractional flow reserve (FFR), an invasive physiologic metric, provides a quantitative assessment of the hemodynamic relevance of coronary stenoses. Multiple large-scale randomized controlled trials have demonstrated that percutaneous coronary intervention (PCI) guided by FFR significantly decreases adverse clinical events compared to strategies based solely on angiography or medical therapy (3, 4). Reflecting this evidence, the European Society of Cardiology (ESC) guidelines now advocate the use of FFR in evaluating intermediate coronary lesions and shaping interventional strategies (5).

Beyond physiologic assessment, intravascular ultrasound (IVUS) plays a pivotal role in optimizing PCI procedures. By offering high-resolution visualization of vessel morphology, plaque composition, and stent-related parameters such as expansion and apposition, IVUS enhances procedural precision (6). Several clinical trials have indicated that IVUS-guided PCI yields superior outcomes compared to angiographic guidance alone (7, 8). Nevertheless, IVUS has conventionally been regarded as an adjunct imaging tool rather than a primary determinant in revascularization strategy formulation.

Myocardial bridging (MB), a congenital coronary anomaly characterized by an intramyocardial course of a coronary segment rather than the typical epicardial trajectory, has gained renewed attention (9). Once considered benign, MB is now recognized to be linked with a spectrum of clinical manifestations, including myocardial ischemia (10), coronary vasospasm (11), acute coronary syndrome(ACS) (12), exercise-induced arrhythmias (13), myocardial stunning (14), syncope, and even sudden cardiac death (15). Notably, MB is frequently accompanied by atherosclerotic plaque accumulation in the proximal segment of the tunneled artery. These plaques are often unstable and susceptible to rupture, thereby elevating the risk of thrombosis and acute coronary events (16).

Interventionally treating MB with stent implantation has produced suboptimal long-term outcomes, primarily due to elevated risks of in-stent restenosis, thrombosis, and mechanical complications such as stent fracture (9). Although surgical interventions such as myotomy or coronary artery bypass grafting are available, their associated procedural risks remain high (17). Consequently, medical management is generally preferred for most MB cases. However, in patients with concurrent MB and proximal intermediate coronary stenosis, the resultant hemodynamic interplay may mimic the effects of serial lesions, significantly impairing myocardial perfusion. MB has been shown to delay diastolic coronary flow, particularly in individuals with diastolic dysfunction (18). This temporal overlap during the cardiac cycle can intensify ischemic burden, suggesting the need for a more proactive therapeutic approach. Nonetheless, data on optimal revascularization strategies in such scenarios remain sparse.

This study aims to evaluate the clinical value of FFR in guiding therapy for patients with moderate MB and proximal intermediate coronary stenosis with documented ischemia. An integrated approach combining CAG, FFR, and IVUS is applied, with PCI performed under IVUS guidance in patients with FFR ≤ 0.80.

## 2 Materials and methods

### 2.1 Study participants

This retrospective, single-center observational study was carried out in the Department of Cardiology at Xiangtan Central Hospital. Patients consecutively admitted between June 2017 and December 2022 for elective PCI, with symptoms of severe exertional angina, confirmed myocardial ischemia, or non-emergent ACS, were screened for eligibility. Inclusion criteria required CAG confirmation of isolated moderate MB in the mid-left anterior descending (LAD) artery—characterized by systolic compression between 50% and 70%—in conjunction with proximal intermediate coronary stenosis. A total of 238 patients met the selection criteria and provided informed consent. Participants were categorized into two groups based on FFR outcomes: those with FFR > 0.80 (*n* = 96) received optimal medical therapy alone, while patients with FFR ≤ 0.80 (*n* = 142) underwent IVUS-guided PCI with implantation of at least one drug-eluting stent (DES) in the proximal lesion, followed by routine medical management. Clinical demographics, comorbidities, laboratory parameters, imaging findings from CAG and IVUS, procedural details, and follow-up outcomes were retrospectively retrieved from the institutional electronic records and imaging archive. The study adhered to the Declaration of Helsinki (2013 revision) and was approved by the Xiangtan Central Hospital Ethics Committee (Approval No. X2019432). Written informed consent was obtained from all participants; verbal consent was documented per institutional protocols when written consent could not be acquired.

**Figure 1:**
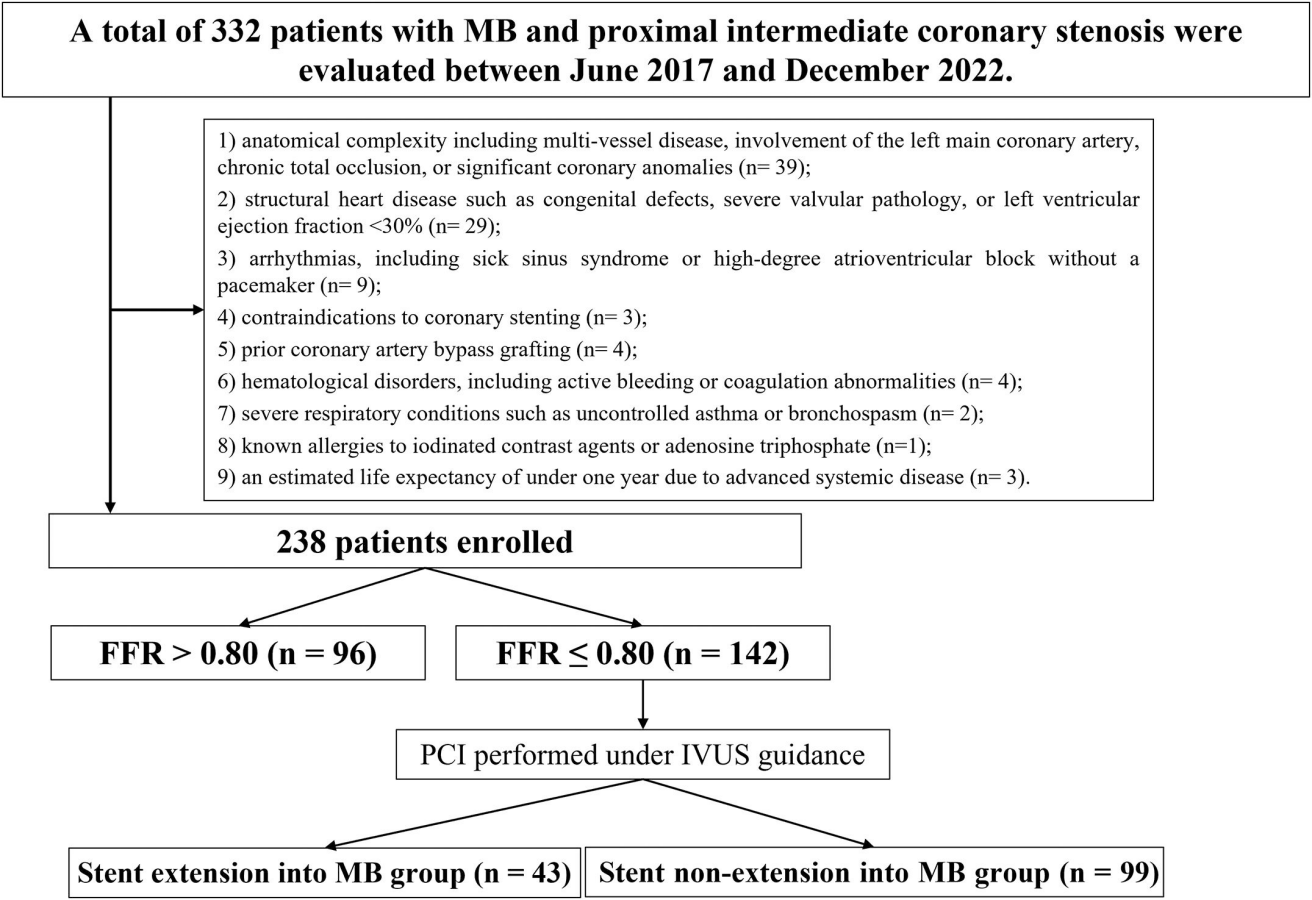
Study flowchart. MB, myocardial bridging; IVUS, intravascular ultrasound; PCI, percutaneous coronary intervention; FFR, fractional flow reserve.

Eligibility was based on diagnostic standards set by the American College of Cardiology (ACC) and the American Heart Association (AHA) for unstable angina (UA), non–ST-segment elevation myocardial infarction (NSTEMI), ST-segment elevation myocardial infarction (STEMI), or stable coronary artery disease (CAD) with objective ischemia, and all participants were scheduled for elective PCI (19). Additional criteria included: (1) angiographic evidence of moderate MB, defined as transient systolic narrowing of 50%–70% in multiple views with diastolic luminal recovery (i.e., the classical “milking effect”) (20); (2) proximal intermediate coronary stenosis, defined as 50%–70% luminal narrowing within 10 mm upstream of the bridged segment (21); and (3) single-vessel disease, with all other lesions either showing <20% stenosis or having undergone previous revascularization. Myocardial ischemia was confirmed by at least one of the following: transient ST-segment depression during angina episodes; a positive treadmill test accompanied by typical symptoms and ≥2 min ST-segment depression; echocardiographic evidence of regional wall motion abnormalities; or abnormal perfusion results on stress nuclear myocardial imaging (22). Exclusion criteria were as follows: (1) anatomical complexity including multi-vessel disease, involvement of the left main coronary artery, chronic total occlusion, or significant coronary anomalies; (2) structural heart disease such as congenital defects, severe valvular pathology, or left ventricular ejection fraction <30%; (3) arrhythmias, including sick sinus syndrome or high-degree atrioventricular block without a pacemaker; (4) contraindications to coronary stenting; (5) prior coronary artery bypass grafting (CABG); (6) hematological disorders, including active bleeding or coagulation abnormalities; (7) severe respiratory conditions such as uncontrolled asthma or bronchospasm; (8) known allergies to iodinated contrast agents or adenosine triphosphate (ATP); and (9) an estimated life expectancy of under one year due to advanced systemic disease (Figure 1).

### 2.2 FFR measurement

FFR was measured using a standard protocol with a PressureWire™ Certus™ pressure guidewire (Abbott Vascular, Santa Clara, CA, USA) connected to a dedicated analyzer system. The pressure wire was advanced 3–5 cm distal to the MB segment after intracoronary nitroglycerin administration (200 μg). Maximal hyperemia was induced with intravenous adenosine triphosphate (ATP, 140 μg/kg·min), and FFR was calculated as the ratio of mean distal coronary pressure (Pd) to aortic pressure (Pa) during stable hyperemia. PCI was performed for FFR ≤ 0.80 and deferred otherwise. Care was taken to avoid wire contact with the vessel wall and to ensure proper alignment distal to the MB (23).

### 2.3 PCI procedures

All PCI procedures were performed by experienced interventional cardiologists, with procedural plans tailored according to the operator's clinical judgment. IVUS was routinely utilized to characterize lesion morphology and to identify appropriate stent landing zones. When indicated, lesion preparation techniques were employed prior to stent deployment. Stents were implanted in reference segments where the plaque burden, as assessed by IVUS, was less than 50%. In situations where the MB segment was located distal to the target lesion, stent deployment within the bridged segment was generally avoided. Nonetheless, when significant dissection extended into the MB or when severe atherosclerotic involvement was present directly adjacent to the MB, stent extension into the bridged region was permitted at the operator's discretion. Technical success was defined by achieving restored antegrade coronary flow corresponding to Thrombolysis in Myocardial Infarction (TIMI) grade 3, along with a residual diameter stenosis of less than 30% in the treated segment. Total procedural duration was measured from the time of vascular access to the final withdrawal of the guiding catheter.

### 2.4 Periprocedural pharmacotherapy

All patients were administered standard dual antiplatelet therapy (DAPT), comprising aspirin (100 mg/day) and clopidogrel (75 mg/day) for at least seven days prior to the procedure. In preparation for the intervention, a loading dose was given 24 h in advance, consisting of aspirin (300 mg) and either clopidogrel (300 mg) or ticagrelor (180 mg), selected according to the treating physician's clinical judgment. Post-PCI, DAPT was continued for 12 months using aspirin (100 mg/day) in combination with clopidogrel (75 mg/day). Additional pharmacologic therapies—including statins, beta-adrenergic blockers, angiotensin-converting enzyme inhibitors (ACEIs) or angiotensin receptor blockers (ARBs), and nitrates—were prescribed individually based on each patient's clinical status and comorbid conditions.

### 2.5 IVUS imaging and analysis

As per institutional standard practice, IVUS imaging was systematically performed in all patients undergoing PCI (FFR ≤ 0.80 group) prior to stent implantation. No patient in the PCI group was excluded for not receiving IVUS. After guidewire placement, 100–200 μg of intracoronary nitroglycerin was administered to reduce vasospasm and improve image quality. The IVUS catheter was advanced distal to the target lesion and withdrawn proximally under fluoroscopic guidance at a controlled rate of 0.5–1.0 mm/s. All imaging sequences were recorded and archived for offline analysis. Quantitative measurements were conducted using QIvus® software (Medis, Leiden, the Netherlands) by two independent observers blinded to clinical data. Discrepancies were resolved by a senior adjudicator. Standardized acquisition and analysis protocols were applied throughout to ensure consistency. MB was defined as an intramyocardial segment of an epicardial artery showing systolic compression and encasement by echolucent muscular tissue on IVUS (24). The following parameters were assessed: minimum lumen area (MLA) at maximal narrowing, plaque burden at the MLA site, maximum MB thickness, total MB length, and diastolic vessel restriction, calculated as (1−diastolic vessel area/interpolated reference area) (18). IVUS guidance was used to ensure stent placement within segments with <50% plaque burden. Stent expansion was assessed as the ratio of the minimum stent area (MSA) to the average reference lumen area of adjacent segments. Stent extension into the MB segment was generally avoided unless clinically necessary, such as in cases of extensive proximal dissection. All IVUS measurements were obtained during presumed end-diastole to ensure consistency. Final anatomical assessments, incorporating both IVUS and angiographic data, were independently reviewed by two experienced interventional cardiologists (X.W. and H.H.) blinded to treatment allocation. Inter- and intra-observer agreement was high, with κ values of 0.89 and 0.92, respectively.

### 2.6 Follow-up and clinical outcomes

Patients underwent scheduled follow-up evaluations at 1, 6, and 12 months post-discharge, and annually thereafter. Follow-up information was collected through a combination of outpatient clinic visits, electronic hospital records, and structured telephone interviews. When necessary, additional verification was conducted via communication with referring physicians or by consulting national mortality registries. The primary study endpoint was the incidence of major adverse cardiovascular events (MACE), defined as a composite outcome including cardiac death, target vessel myocardial infarction (MI), clinically indicated target lesion revascularization (TLR), rehospitalization for recurrent anginal symptoms, definite or probable in-stent thrombosis (IST), acute heart failure, or life-threatening arrhythmias. These events were classified according to standardized criteria established by the Academic Research Consortium (25).

### 2.7 Statistical analysis

Statistical analyses were conducted using IBM SPSS Statistics version 26.0 (IBM Corp., Armonk, NY, USA). The Kolmogorov–Smirnov test was applied to evaluate the normality of distribution for continuous variables. Data with a normal distribution were presented as mean ± standard deviation (SD) and compared using independent-samples *t*-tests. For variables not following a normal distribution, data were expressed as median with interquartile range (IQR) and analyzed using the Mann–Whitney *U* test. Categorical variables were summarized as frequencies and percentages, with comparisons made using Pearson's *χ*^2^ test or Fisher's exact test, depending on data characteristics. To determine independent predictors of MACE, variables that reached statistical significance in univariate analysis were included in a multivariate Cox proportional hazards regression model employing backward stepwise elimination. Time-to-event outcomes were analyzed using Kaplan–Meier survival curves, and differences between groups were evaluated by the log-rank test. A two-sided *p*-value of less than 0.05 was considered indicative of statistical significance. Additionally, among patients with FFR ≤ 0.80 who underwent PCI, a predefined subgroup analysis was performed to compare clinical outcomes between those who received stent extension into the MB segment and those who did not.

## 3 Results

A total of 238 patients were enrolled, with 96 in the FFR > 0.80 group and 142 in the FFR ≤ 0.80 group. Baseline demographic and clinical features were generally well balanced between the two cohorts. No significant differences were observed in age, sex, cardiovascular risk factors (including hypertension, diabetes mellitus, and dyslipidemia), or background pharmacological treatment (Table 1).

**Table 1:** Baseline characteristics.

Variables	All (*n* = 238)	FFR > 0.80 (*n* = 96)	FFR ≤ 0.80 (*n* = 142)	*P* value
Age, years	63.06 (60.45, 66.02)	62.94 (60.41, 65.82)	63.32 (60.47, 66.14)	0.487
Male, *n* %	143 (60.1%)	57 (59.4%)	86 (60.6%)	0.961
Prior hypertension, *n* %	114 (47.9%)	44 (45.8%)	70 (49.3%)	0.694
Prior hyperlipidemia, *n* %	89 (37.4%)	33 (34.4%)	56 (39.4%)	0.512
Prior diabetes mellitus, *n* %	66 (27.7%)	30 (31.2%)	36 (25.4%)	0.395
Prior stroke, *n* %	13 (5.5%)	6 (6.2%)	7 (4.9%)	0.881
Smoking, *n* %	114 (47.9%)	48 (50.0%)	66 (46.5%)	0.688
Chronic kidney diseasea^a^, *n* %	12 (5.0%)	7 (7.3%)	5 (3.5%)	0.316
Peripheral artery disease, *n* %	30 (12.6%)	13 (13.5%)	17 (12.0%)	0.873
Prior myocardial infarction, *n* %	68 (28.6%)	24 (25.0%)	44 (31.0%)	0.391
Prior PCI, *n* %	29 (12.2%)	10 (10.4%)	19 (13.4%)	0.628
Laboratory biomarkers				
Platelet count, 10^9^ /L	249.80 (233.41, 264.70)	248.08 (231.41, 264.82)	251.41 (234.41, 264.70)	0.626
TG, mmol/L	1.88 (1.68, 2.02)	1.83 (1.67, 1.99)	1.91 (1.71, 2.04)	0.113
TC, mmol/L	5.32 (5.16, 5.53)	5.31 (5.19, 5.52)	5.33 (5.16, 5.54)	0.857
HDL, mmol/L	1.26 (1.16, 1.37)	1.28 (1.18, 1.36)	1.26 (1.16, 1.38)	0.742
LDL, mmol/L	3.36 (3.21, 3.50)	3.36 (3.15, 3.50)	3.35 (3.24, 3.50)	0.428
Lp(a), mg/L	197.80 (171.03, 224.06)	197.68 (164.96, 217.10)	199.03 (173.23, 226.02)	0.301
AST, U/L	113.30 (98.57, 130.96)	113.30 (101.54, 129.07)	113.27 (97.54, 133.45)	0.922
ALT, U/L	48.84 (40.33, 56.08)	48.66 (39.58, 54.40)	49.71 (40.75, 56.89)	0.507
TBIL, umol/L	16.48 (15.11, 17.83)	16.48 (14.57, 17.44)	16.48 (15.31, 18.03)	0.073
Uric acid, umol/L	475.31 (438.44, 517.02)	471.94 (437.88, 513.18)	475.86 (442.11, 518.33)	0.358
Scr, umol/L	88.92 (85.62, 92.83)	89.81 (86.18, 94.26)	88.72 (85.44, 92.69)	0.212
eGFR, mL/min per 1.732 m^2^	99.56 (92.65, 108.89)	99.15 (90.79, 106.66)	99.71 (93.39, 110.28)	0.366
Pharmacologic therapy				
DAPT, *n* %	238 (100.0%)	96 (100.0%)	142 (100.0%)	1.000
Statins, *n* %	221 (92.9%)	92 (95.8%)	129 (90.8%)	0.226
ACEI or ARB, *n* %	140 (58.8%)	59 (61.5%)	81 (57.0%)	0.585
Beta-blockers, *n* %	194 (81.5%)	74 (77.1%)	120 (84.5%)	0.307
Aldosterone antagonists, *n* %	40 (16.8%)	15 (15.6%)	25 (17.6%)	0.822
Nitrates, *n* %	9 (3.8%)	3 (3.1%)	6 (4.2%)	0.927
Calcium channel blockers, *n* %	29 (12.2%)	10 (10.4%)	19 (13.4%)	0.628

Continuous variables were expressed as median (interquartile range). Categorical variables were expressed as number (percentage).

FFR, fractional flow reserve; PCI, percutaneous coronary intervention; DAPT, dual antiplatelet therapy; ACEI, angiotensin-converting enzyme inhibitor; ARB, angiotensin-receptor blocker; TG, triglycerides; TC, total cholesterol; HDL, high-density lipoprotein; LDL, low-density lipoprotein; Lp(a), lipoprotein(a); AST, aspartate aminotransferase; ALT, alanine aminotransferase; TBIL, total bilirubin; Scr, serum creatinine; eGFR, estimated glomerular filtration rate.

a Estimated glomerular filtration rate <60 mL/min/1.73 m^2^ using the Modification of Diet in Renal Disease study equation.

Angiographic and procedural characteristics were also comparable across groups. Parameters such as proximal stenosis severity, MB segment length, degree of systolic compression, and the distance from the proximal stenosis to the MB did not differ significantly. As expected, the FFR ≤ 0.80 group exhibited a significantly lower baseline FFR value (0.71 ± 0.03 vs. 0.88 ± 0.03, *P* < 0.001). Within this group, 43 patients (30.3%) underwent stent extension into the MB segment (Table 2).

**Table 2:** Angiographic and procedural findings.

Variables	All (*n* = 238)	FFR > 0.80 (*n* = 96)	FFR ≤ 0.80 (*n* = 142)	*P* value
Pre-PCI FFR	0.78 ± 0.09	0.88 ± 0.03	0.71 ± 0.03	<0.001
Proximal stenosis severity, %	66.19 ± 6.16	65.81 ± 6.41	66.45 ± 5.95	0.357
Lesion length >20 mm, *n* %	68 (28.6%)	26 (27.1%)	42 (29.6%)	0.785
Lesion length, mm	36.24 ± 3.60	35.92 ± 3.66	36.45 ± 3.54	0.298
MB length, mm	11.04 ± 4.04	10.56 ± 4.83	11.36 ± 3.35	0.170
Systolic compression severity of the bridged segment, %	63.95 ± 7.08	63.28 ± 7.07	64.41 ± 7.02	0.175
Distance from proximal stenosis to the MB, mm	14.75 ± 3.05	15.20 ± 3.18	14.44 ± 2.90	0.090
Calcification, *n* %	23 (9.7%)	10 (10.4%)	13 (9.2%)	0.920
Calcium length, mm	10.04 ± 0.54	10.06 ± 0.53	10.02 ± 0.55	0.769
Lesion bend, *n* %	25 (10.5%)	9 (9.4%)	16 (11.3%)	0.801
Post-PCI in-segment^a^				
Reference vessel diameter, mm	–	–	3.22 ± 0.53	–
Minimum lumen diameter, mm	–	–	2.58 ± 0.19	–
Diameter stenosis, %	–	–	22.23 ± 3.30	–
Post-PCI distal vessel				
Reference vessel diameter, mm	–	–	1.95 ± 1.03	–
Minimum lumen diameter, mm	–	–	1.17 ± 0.31	–
Diameter stenosis, %	–	–	29.29 ± 3.73	–
Procedural findings				
Total stent length, mm	–	–	39.30 ± 5.93	–
Post-PCI FFR	–	–	0.89 ± 0.04	–
Stent extension into MB	–	–	43 (30.3)	–
Maximum device diameter, mm	–	–	3.28 ± 2.46	–
Maximum balloon inflation pressure, atm	–	–	18.58 ± 3.00	–
Procedure time, min	–	–	45.99 ± 6.94	–
Radiation exposure dose, Gy	–	–	1.97 ± 0.30	–
Contrast media volume, ml	–	–	271.11 ± 15.43	–

Continuous variables were expressed as mean ± SD, or median (interquartile range). Categorical variables were expressed as number (percentage).

FFR, fractional flow reserve; PCI, percutaneous coronary intervention; MB, myocardial bridging.

a In-segment includes stent and 5 mm proximal and distal reference from each stent edge.

**Figure 2:**
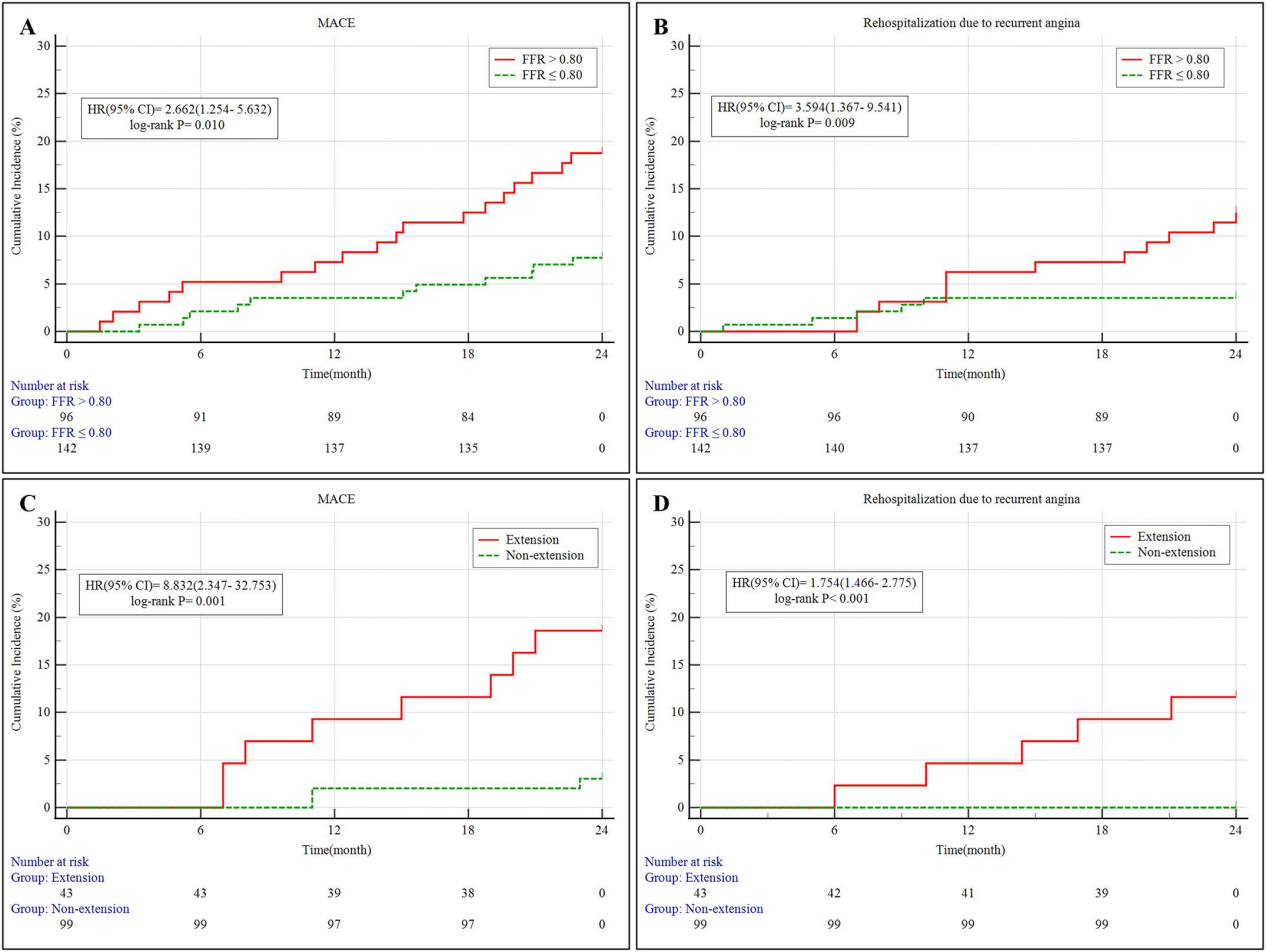
**(A,B)** Kaplan–Meier survival curves of MACE and rehospitalization due to recurrent angina in patients stratified by FFR for 2 years. **(C,D)** Kaplan–Meier survival curves of MACE and rehospitalization due to recurrent angina in the PCI subgroup (FFR ≤ 0.80) stratified by stent extension into MB for 2 years. MB, myocardial bridging; MACE, major adverse cardiovascular events; 95% CI, 95% confidence intervals; HR, hazard ratio; PCI, percutaneous coronary intervention; FFR, fractional flow reserve;.

At two-year follow-up, the FFR > 0.80 group experienced a higher rate of MACE (18.8% vs. 7.7%, *P* = 0.019), primarily driven by increased rehospitalization for recurrent angina (12.5% vs. 3.5%, *P* = 0.017), compared to those in the FFR ≤ 0.80 group. Other individual endpoints, including cardiac mortality, MI, TLR, acute heart failure, and malignant arrhythmias, showed no statistically significant differences (Table 3; Figures 2A,B).

**Table 3:** 2-year clinical outcomes.

Variables	All (*n* = 238)	FFR > 0.80 (n = 96)	FFR ≤ 0.80 (*n* = 142)	OR	95% CI	*P* value
MACE, *n* %	29 (12.2%)	18 (18.8%)	11 (7.7%)	2.742	1.233–6.121	0.019
Cardiac death, *n* %	3 (1.3%)	2 (2.1%)	1 (0.7%)	3.154	0.268–13.557	0.731
Target vessel MI, *n* %	5 (2.1%)	3 (3.1%)	2 (1.4%)	2.256	0.370–9.773	0.656
Clinically driven TLR, *n* %	8 (3.4%)	6 (6.2%)	2 (1.4%)	4.661	0.921–8.630	0.095
Rehospitalization due to recurrent angina, *n* %	17 (7.1%)	12 (12.5%)	5 (3.5%)	3.914	1.331–11.504	0.017
In-stent thrombosis, *n* %	–	–	1 (0.7%)	–	–	–
Acute heart failure, *n* %	6 (2.5%)	4 (4.2%)	2 (1.4%)	3.043	0.546–16.956	0.362
Malignant arrhythmias, *n* %	3 (1.3%)	2 (2.1%)	1 (0.7%)	2.532	0.268–5.175	0.731

Categorical variables were expressed as number (percentage).

FFR, fractional flow reserve; MACE, major adverse cardiovascular events; MI, myocardial infarction; TLR, target lesion revascularizatio; OR, odds ratios; CI, confidence interval;.

Multivariate Cox regression identified PCI as an independent protective factor against MACE [hazard ratio [HR] = 0.526, 95% confidence interval (CI): 0.247–0.969, *P* = 0.034]. Conversely, stent extension into the MB was independently associated with a higher risk of MACE (HR = 2.632, 95% CI: 1.778–3.674, *P* = 0.016). Other variables, such as diabetes status, degree of proximal stenosis, and MB compression severity, were not independently predictive (Table 4).

**Table 4:** Univariate and multivariate Cox regression analyses showing independent predictors of MACE.

Variables	Univariate analysis			Multivariate analysis		
	HR	95% CI	*P* value	HR	95% CI	*P* value
Age (per 10 years)	1.185	0.903–1.553	0.227			
Male sex	1.154	0.702–1.725	0.656			
Hypertension	1.252	0.825–1.899	0.315			
Diabetes mellitus	1.652	1.054–2.586	0.033	1.383	0.848–2.156	0.121
Proximal stenosis severity (%)	1.034	1.014–1.126	0.026	1.122	0.623–2.012	0.704
Systolic compression of MB (%)	1.043	1.015–1.074	0.013	1.175	0.543–2.435	0.701
MB length (mm)	1.023	0.948–1.078	0.115			
FFR ≤ 0.80	1.645	0.328–2.649	0.853			
PCI performed	0.484	0.244–0.953	0.035	0.526	0.247–0.969	0.034
Stent extension into MB	1.483	1.401–2.174	0.043	2.632	1.778–3.674	0.016

MACE, major adverse cardiovascular events; HR, hazard ratios; CI, confidence interval; MB, myocardial bridging; FFR, fractional flow reserve.

Within the PCI-treated subgroup (*n* = 142), patients were further divided based on whether the stent extended into the MB. The MB-stent group comprised 43 patients (30.3%), while 99 patients (69.7%) received stents without crossing the MB. Baseline variables were generally similar between the two groups (Table 5). Although not statistically significant, there was a trend toward fewer male patients (48.8% vs. 65.7%, *P* = 0.059), and a lower prevalence of hypertension (37.2% vs. 54.5%, *P* = 0.057) and hyperlipidemia (27.9% vs. 44.4%, *P* = 0.063) in the MB-stent group.

**Table 5:** Baseline characteristics in the PCI subgroup (FFR ≤ 0.80) stratified by stent extension into MB.

Variables	All (*n* = 142)	Stent extension into MB group (*n* = 43)	Stent non-extension group (*n* = 99)	*P* value
Age, years	63.03 (61.30, 64.73)	62.82 (61.24, 64.53)	63.10 (61.38, 64.85)	0.732
Male, *n* %	86 (60.6%)	21 (48.8%)	65 (65.7%)	0.059
Prior hypertension, *n* %	70 (49.3%)	16 (37.2%)	54 (54.5%)	0.057
Prior hyperlipidemia, *n* %	56 (39.4%)	12 (27.9%)	44 (44.4%)	0.063
Prior diabetes mellitus, *n* %	36 (25.4%)	7 (16.3%)	29 (29.3%)	0.101
Prior stroke, *n* %	7 (4.9%)	0 (0.0%)	7 (7.1%)	0.073
Smoking, *n* %	66 (46.5%)	15 (34.9%)	51 (51.5%)	0.067
Chronic kidney diseasea^a^, *n* %	5 (3.5%)	0 (0.0%)	5 (5.1%)	0.133
Peripheral artery disease, *n* %	17 (12.0%)	2 (4.7%)	15 (15.2%)	0.076
Prior myocardial infarction, *n* %	44 (31.0%)	9 (20.9%)	35 (35.4%)	0.087
Prior PCI, *n* %	19 (13.4%)	3 (7.0%)	16 (16.2%)	0.139
Laboratory biomarkers				
Platelet count, 10^9^/L	251.41 (234.41, 264.70)	252.27 (241.54, 262.68)	250.45 (234.06, 266.26)	0.911
TG, mmol/L	1.91 (1.71, 2.04)	1.93 (1.76, 2.08)	1.87 (1.71, 2.01)	0.257
TC, mmol/L	5.33 (5.16, 5.54)	5.37 (5.16, 5.48)	5.33 (5.15, 5.56)	0.657
HDL, mmol/L	1.26 (1.16, 1.38)	1.29 (1.18, 1.38)	1.25 (1.15, 1.38)	0.374
LDL, mmol/L	3.35 (3.24, 3.50)	3.34 (3.22, 3.46)	3.38 (3.25, 3.50)	0.505
Lp(a), mg/L	199.03 (173.23, 226.02)	200.82 (186.67, 230.84)	197.42 (169.72, 225.09)	0.152
AST, U/L	113.27 (97.54, 133.45)	117.85 (103.13, 130.67)	112.77 (96.76, 134.95)	0.715
ALT, U/L	49.71 (40.75, 56.89)	50.06 (40.44, 55.06)	49.43 (41.52, 56.96)	0.834
TBIL, umol/L	16.48 (15.31, 18.03)	16.44 (15.36, 18.03)	16.50 (15.28, 18.03)	0.862
Uric acid, umol/L	475.86 (442.11, 518.33)	473.81 (437.92, 516.80)	477.21 (445.24, 519.76)	0.622
Scr, umol/L	88.72 (85.44, 92.69)	88.74 (86.42, 93.79)	88.71 (84.05, 92.36)	0.282
eGFR, mL/min per 1.732 m^2^	99.71 (93.39, 110.28)	99.40 (93.33, 107.80)	100.11 (93.57, 111.10)	0.569
Pharmacologic therapy				
DAPT, *n* %	142 (100.0%)	43 (100.0%)	99 (100.0%)	1.000
Statins, *n* %	129 (90.8%)	41 (95.3%)	88 (88.9%)	0.223
ACEI or ARB, *n* %	81 (57.0%)	24 (55.8%)	57 (57.6%)	0.848
Beta-blockers, *n* %	120 (84.5%)	40 (93.0%)	80 (80.8%)	0.103
Aldosterone antagonists, *n* %	25 (17.6%)	8 (18.6%)	17 (17.2%)	0.840
Nitrates, *n* %	6 (4.2%)	2 (4.7%)	4 (4.0%)	0.919
Calcium channel blockers, *n* %	19 (13.4%)	6 (14.0%)	13 (13.1%)	0.898

Continuous variables were expressed as median (interquartile range). Categorical variables were expressed as number (percentage).

FFR, fractional flow reserve; PCI, percutaneous coronary intervention; DAPT, dual antiplatelet therapy; ACEI, angiotensin-converting enzyme inhibitor; ARB, angiotensin-receptor blocker; TG, triglycerides; TC, total cholesterol; HDL, high-density lipoprotein; LDL, low-density lipoprotein; Lp(a), lipoprotein(a); AST, aspartate aminotransferase; ALT, alanine aminotransferase; TBIL, total bilirubin; Scr, serum creatinine; eGFR, estimated glomerular filtration rate; MB, myocardial bridging.

a Estimated glomerular filtration rate <60 mL/min/1.73 m^2^ using the Modification of Diet in Renal Disease study equation.

As presented in Table 6, patients receiving MB stenting had longer lesion lengths (37.35 ± 3.24 mm vs. 36.06 ± 3.58 mm, *P* = 0.038), longer MB segments (12.26 ± 3.37 mm vs. 10.96 ± 3.27 mm, *P* = 0.036), and more severe systolic compression (67.71 ± 5.60% vs. 62.90 ± 7.01%, *P* < 0.001). Furthermore, the distance from the proximal lesion to the MB was significantly shorter in the MB-stent group (13.63 ± 2.88 mm vs. 14.79 ± 2.84 mm, *P* = 0.030).

**Table 6:** Angiographic and procedural findings in the PCI subgroup (FFR ≤ 0.80) stratified by stent extension into MB.

Variables	All (*n* = 142)	Stent extension into MB group (*n* = 43)	Stent non-extension group (*n* = 99)	*P* value
Pre-PCI FFR	0.71 ± 0.03	0.70 ± 0.03	0.72 ± 0.03	0.057
Proximal stenosis severity, %	66.45 ± 5.95	66.87 ± 5.88	66.27 ± 5.97	0.587
Lesion length >20 mm, *n* %	42 (29.6%)	20 (46.5%)	22 (22.2%)	0.006
Lesion length, mm	36.45 ± 3.54	37.35 ± 3.24	36.06 ± 3.58	0.038
MB length, mm	11.36 ± 3.35	12.26 ± 3.37	10.96 ± 3.27	0.036
Systolic compression severity of the bridged segment, %	64.41 ± 7.02	67.71 ± 5.60	62.90 ± 7.01	<0.001
Distance from proximal stenosis to the MB, mm	14.44 ± 2.90	13.63 ± 2.88	14.79 ± 2.84	0.030
Calcification, *n* %	13 (9.2%)	3 (7.0%)	10 (10.1%)	0.782
Calcium length, mm	10.02 ± 0.55	9.94 ± 0.48	10.05 ± 0.58	0.252
Lesion bend, *n* %	16 (11.3%)	5 (11.6%)	11 (11.1%)	1.000
Post-PCI in-segment^a^				
Reference vessel diameter, mm	3.22 ± 0.53	3.26 ± 0.59	3.20 ± 0.50	0.542
Minimum lumen diameter, mm	2.58 ± 0.19	2.58 ± 0.19	2.58 ± 0.19	0.933
Diameter stenosis, %	22.23 ± 3.30	22.29 ± 2.95	22.20 ± 3.46	0.885
Post-PCI distal vessel				
Reference vessel diameter, mm	1.95 ± 1.03	2.03 ± 1.14	1.91 ± 0.98	0.542
Minimum lumen diameter, mm	1.17 ± 0.31	1.18 ± 0.32	1.17 ± 0.30	0.933
Diameter stenosis, %	29.29 ± 3.73	29.35 ± 3.34	29.26 ± 3.90	0.875
Procedural findings				
Total stent length, mm	39.30 ± 5.93	40.95 ± 6.25	38.58 ± 5.66	0.027
Post-PCI FFR	0.89 ± 0.04	0.89 ± 0.04	0.88 ± 0.04	0.107
Maximum device diameter, mm	3.28 ± 2.46	3.01 ± 2.55	3.39 ± 2.42	0.395
Maximum balloon inflation pressure, atm	18.58 ± 3.00	18.21 ± 3.24	18.74 ± 2.89	0.340
Procedure time, min	45.99 ± 6.94	46.53 ± 7.72	45.76 ± 6.60	0.542
Radiation exposure dose, Gy	1.97 ± 0.30	1.98 ± 0.31	1.97 ± 0.29	0.933
Contrast media volume, ml	271.11 ± 15.43	271.39 ± 13.80	270.98 ± 16.15	0.885

Continuous variables were expressed as mean ± SD. Categorical variables were expressed as number (percentage).

FFR, fractional flow reserve; PCI, percutaneous coronary intervention; MB, myocardial bridging.

a In-segment includes stent and 5 mm proximal and distal reference from each stent edge.

Post-PCI IVUS assessments (Table 7) revealed a higher incidence of dissection (44.2% vs. 19.2%, *P* = 0.003) and dissection extension into the MB (34.9% vs. 9.1%, *P* < 0.001) in the MB-stent group. Other IVUS parameters, including plaque burden, calcification, and stent expansion, were similar between groups.

**Table 7:** Intravascular ultrasound findings in the PCI subgroup (FFR ≤ 0.80) stratified by stent extension into MB.

Variables	All (*n* = 142)	Stent extension into MB group (*n* = 43)	Stent non-extension group (*n* = 99)	*P* value
Lesion length, mm	38.74 ± 5.65	39.75 ± 6.25	38.30 ± 5.35	0.160
Maximum plaque burden, %	83.78 ± 5.24	83.84 ± 5.43	83.76 ± 5.18	0.933
Calcification in lesion, *n* %	33 (23.2%)	11 (25.6%)	22 (22.2%)	0.826
Maximum arc of calcium,°	123.58 ± 20.74	123.97 ± 18.55	123.42 ± 21.72	0.885
Dissection, *n* %	38 (26.8%)	19 (44.2%)	19 (19.2%)	0.003
Dissection extended into an MB, *n* %	24 (16.9%)	15 (34.9%)	9 (9.1%)	<0.001
Reference minimum lumen area, mm^2^	3.68 ± 1.35	3.82 ± 1.50	3.62 ± 1.29	0.417
Reference maximum plaque burden, %	58.05 ± 3.37	58.48 ± 3.30	57.86 ± 3.39	0.313
MB segment				
Distance from LAD ostium to MB, mm	34.15 ± 6.91	34.77 ± 7.27	33.88 ± 6.78	0.485
Total MB length, mm	10.83 ± 2.43	12.21 ± 2.40	10.23 ± 2.19	<0.001
Maximum thickness of MB, mm	0.48 ± 0.08	0.48 ± 0.08	0.47 ± 0.08	0.433
Diastolic vessel area at max compression site, mm^2^	4.34 ± 0.61	4.32 ± 0.53	4.35 ± 0.64	0.792
Diastolic vessel restriction, %	19.57 ± 4.29	18.60 ± 4.48	19.99 ± 4.17	0.077
Minimum lumen area, mm^2^	2.41 ± 0.60	2.39 ± 0.67	2.41 ± 0.57	0.828
Plaque burden at minimum lumen area site, %	41.15 ± 4.05	41.44 ± 4.04	41.02 ± 4.07	0.569
Postprocedure findings				
MSA, mm^2^	5.43 ± 3.22	5.21 ± 2.57	5.53 ± 3.48	0.588
Stent expansion, %	70.36 ± 3.24	70.60 ± 2.71	70.26 ± 3.45	0.565
Rate of MSA in the MB, when stented, *n* %	71 (50.0%)	21 (48.8%)	50 (50.5%)	0.856

Continuous variables were expressed as mean ± SD. Categorical variables were expressed as number (percentage).

MB, myocardial bridging; MSA, minimum stent area; LAD, left anterior descending artery; FFR, fractional flow reserve; PCI, percutaneous coronary intervention;.

During the 2-year follow-up, MACE occurred significantly more often in patients with MB stent extension (18.6% vs. 3.0%, *P* = 0.001), mainly due to a markedly higher rate of angina-related rehospitalization (11.6% vs. 0%, *P* < 0.001) (Table 8; Figures 2C,D). No significant differences were observed in cardiac death, target vessel MI, or TLR between the subgroups.

**Table 8:** 2-year clinical outcomes in the PCI subgroup (FFR ≤ 0.80) stratified by stent extension into MB.

Variables	All (*n* = 142)	Stent extension into MB group (*n* = 43)	Stent non-extension group (*n* = 99)	OR	95% CI	*P* value
MACE, *n* %	11 (7.7%)	8 (18.6%)	3 (3.0%)	1.717	1.105–4.240	0.001
Cardiac death, *n* %	1 (0.7%)	0 (0.0%)	1 (1.0%)	0.877	0.686–2.122	0.508
Target vessel MI, *n* %	2 (1.4%)	1 (2.3%)	1 (1.0%)	0.944	0.839–1.061	0.541
Clinically driven TLR, *n* %	2 (1.4%)	1 (2.3%)	1 (1.0%)	1.000	0.684–1.463	0.541
Rehospitalization due to recurrent angina, *n* %	5 (3.5%)	5 (11.6%)	0 (0.0%)	1.026	1.004–2.049	<0.001
In-stent thrombosis, *n* %	1 (0.7%)	1 (2.3%)	0 (0.0%)	0.981	0.925–1.040	0.127
Acute heart failure, *n* %	2 (1.4%)	0 (0.0%)	2 (2.0%)	0.996	0.976–1.016	0.347
Malignant arrhythmias, *n* %	1 (0.7%)	1 (2.3%)	0 (0.0%)	1.019	0.982–1.056	0.127

Categorical variables were expressed as number (percentage).

FFR, fractional flow reserve; MACE, major adverse cardiovascular events; MI, myocardial infarction; TLR, target lesion revascularizatio; PCI, percutaneous coronary intervention; MB, myocardial bridging; OR, odds ratios; CI, confidence interval;.

**Figure 3:**
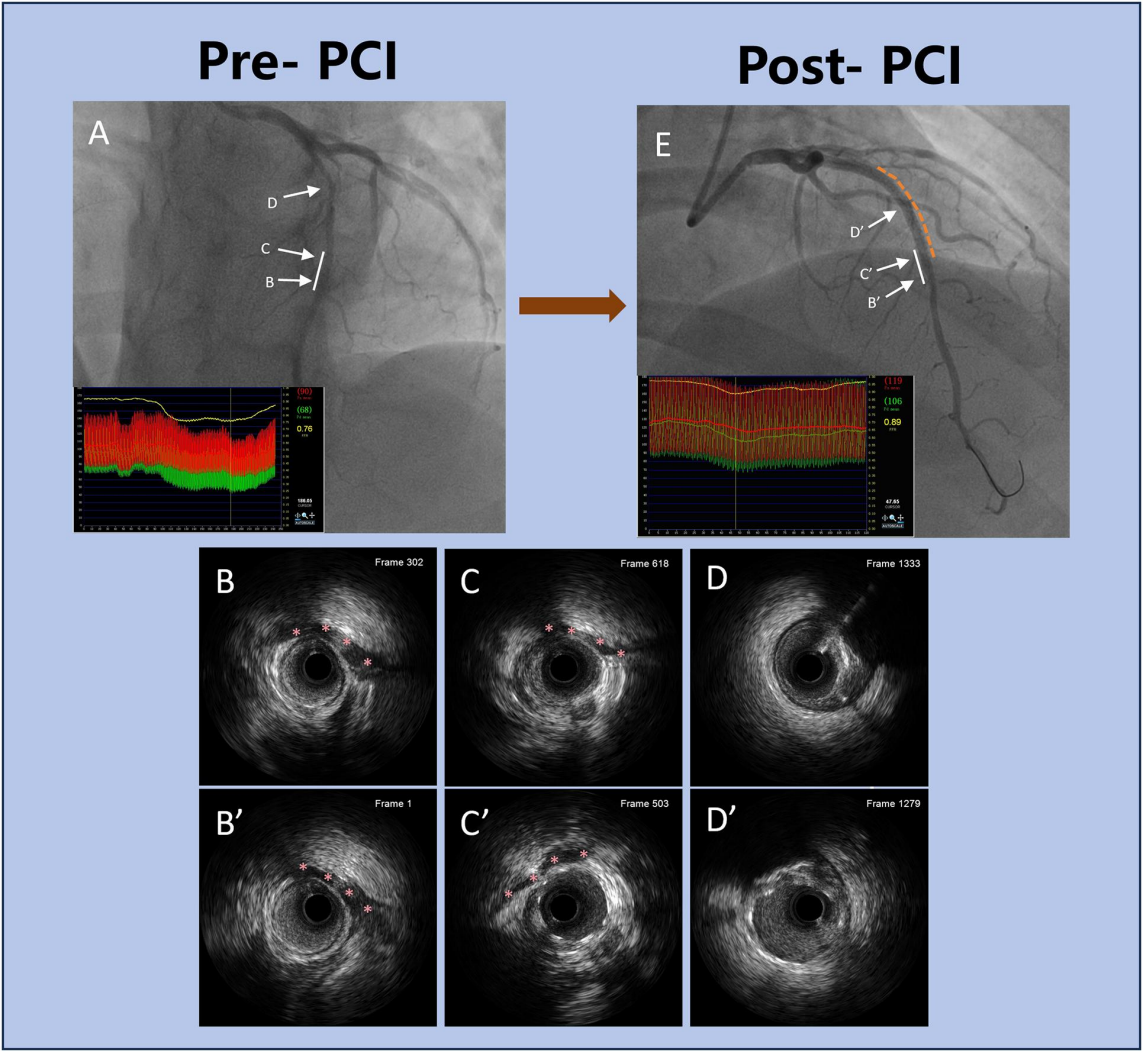
Representative clinical case of PCI in a patient with proximal LAD stenosis and MB. **(A)** Pre-PCI coronary angiography showing moderate proximal LAD stenosis adjacent to a MB. The white solid line indicates the MB segment. The co-registered FFR tracing reveals a nadir value of 0.76 distal to the MB, indicating functionally significant ischemia. **(B–D)** Corresponding pre-PCI IVUS images at levels **(B–D)**. Red asterisks in **(B,C)** mark the MB segment identified on IVUS, showing systolic compression and plaque burden. **(E)** Post-PCI coronary angiography after DES deployment. The white solid line again marks the MB segment, and the yellow dashed line outlines the stented portion of the vessel. FFR improved to 0.89, confirming successful revascularization with improved coronary physiology. (B’–D’) Post-PCI IVUS images at corresponding levels B’–D’. Red asterisks in B’ and C’ indicate the MB segment, showing adequate stent expansion and apposition outside the bridged area, with no evidence of malapposition or dissection. This case highlights the integration of anatomical imaging (angiography, IVUS) and FFR to guide optimal PCI strategy, particularly in lesions involving MB. PCI, percutaneous coronary intervention; LAD, left anterior descending artery; MB, myocardial bridging; FFR, fractional flow reserve; IVUS, intravascular ultrasound; DES, drug-eluting stent.

Multivariate logistic regression (Table 9) identified two independent predictors of stent extension into the MB segment: a shorter distance from the proximal lesion to the MB [odds ratio (OR) = 1.774, 95% CI: 1.542–2.145, *P* = 0.019], and the presence of dissection extending into the MB (OR = 2.435, 95% CI: 2.045–2.745, *P* = 0.041). A representative case of proximal LAD stenosis involving the MB, evaluated by CAG, IVUS, and FFR before and after PCI, is illustrated in Figure 3.

**Table 9:** Univariate and multivariate Cox regression analyses showing independent predictors of stent extension into MB in the PCI subgroup (FFR ≤ 0.80).

Variables	Univariate analysis			Multivariate analysis		
	OR	95% CI	*P* value	OR	95% CI	*P* value
Lesion length >20 mm	1.753	1.363–2.431	0.028	0.862	0.653–1.057	0.792
Lesion length, mm	0.853	0.535–1.066	0.827			
MB length, mm	0.942	0.537–1.164	0.516			
Systolic compression severity of the bridged segment, %	0.923	0.743–1.263	0.452			
Distance from proximal stenosis to the MB, mm	1.413	1.137–1.652	0.018	1.774	1.542–2.145	0.019
Total stent length, mm	0.833	0.424–1.262	0.746			
Dissection	0.862	0.653–1.074	0.792			
Dissection extended into an MB	1.963	1.725–2.356	0.036	2.435	2.045–2.745	0.041

MACE, major adverse cardiovascular events; OR, odds ratios; CI, confidence interval; MB, myocardial bridging; FFR, fractional flow reserve; PCI, percutaneous coronary intervention;.

## 4 Discussion

This study demonstrated that an FFR-guided revascularization strategy was associated with improved long-term clinical outcomes in patients presenting with moderate MB and concomitant proximal intermediate coronary stenosis. Notably, patients with FFR ≤ 0.80 who underwent PCI experienced significantly lower rates of MACE and rehospitalizations for recurrent angina compared to those managed conservatively with medical therapy alone. Multivariate regression confirmed PCI as an independent protective factor against MACE, while stent extension into the MB segment was identified as an independent risk factor. Within the PCI-treated subgroup, patients who received stents extending into the MB region showed a markedly higher incidence of both MACE and angina-related readmissions compared to those whose stents did not involve the bridged segment. Further analysis revealed two independent predictors of elevated MACE risk in this subgroup: a shorter anatomical distance between the proximal stenosis and the MB, and the presence of dissection extending into the bridged segment.

### 4.1 FFR-guided management of moderate myocardial bridging with proximal stenosis

#### 4.1.1 Role of FFR in the functional assessment of MB-related stenosis

Anatomical stenosis does not always reflect the presence or severity of functional ischemia, particularly in dynamic lesions such as MB. This mismatch between anatomical narrowing and physiological impact complicates visual assessment and highlights the necessity of objective functional evaluation. In line with this, the 2018 ESC guidelines recommend FFR as a Class I, Level A tool for assessing intermediate coronary stenoses (5). While FFR is well established in evaluating coronary physiology, its use in MB presents notable challenges. MB causes systolic compression that predominantly impairs diastolic perfusion, yet conventional FFR—derived from mean pressure ratios over the entire cardiac cycle during hyperemia—may fail to capture this diastolic dysfunction. In particular, systolic compression can distort the average gradient without fully reflecting the true ischemic burden. Recent studies suggest that diastolic-specific indices, such as diastolic FFR (dFFR) obtained under dobutamine stress, may more accurately detect MB-related ischemia, with diagnostic thresholds typically set at ≤0.76 (26, 27).

Our findings support this physiological limitation. Patients with FFR > 0.80 who did not undergo PCI demonstrated a higher numerical rate of MACE than those revascularized with FFR ≤ 0.80. This counterintuitive pattern may reflect residual ischemia due to diastolic flow restriction not captured by traditional FFR, especially in cases with coexisting proximal LAD atherosclerosis. Thus, exclusive reliance on conventional FFR in MB-associated disease may lead to undertreatment. These observations underscore the importance of integrating anatomic imaging and phase-specific functional assessment. Tools such as IVUS for structural evaluation, and dFFR or instantaneous wave-free ratio (iFR) for capturing diastolic flow impairment, can provide a more comprehensive assessment. A tailored, anatomy-function integrated strategy may enhance diagnostic precision, improve therapeutic targeting, and ultimately lead to better outcomes in patients with proximal LAD stenosis complicated by MB.

#### 4.1.2 Reconsidering the safety of deferring PCI in FFR > 0.80

PCI deferral in patients with FFR > 0.80 is generally considered safe and aligns with current international guidelines (5). However, in our cohort, patients managed conservatively based on this threshold demonstrated a higher numerical incidence of MACE and angina-related rehospitalizations compared to those who underwent PCI with FFR ≤ 0.80. This paradox challenges the sufficiency of FFR as a standalone tool in guiding therapy for this subset.

Several pathophysiological mechanisms may explain this discrepancy. First, MB introduces dynamic coronary flow alterations via phasic systolic compression and retrograde flow, which can impair diastolic perfusion. Traditional FFR, as a cycle-averaged metric, may overlook these transient disturbances (28, 29). Second, atherosclerotic plaques commonly develop proximal to the MB segment and may remain hemodynamically silent on FFR but still pose a risk for progression or rupture (9, 28). Third, factors such as sympathetic activation or elevated heart rate can further reduce diastolic time, exacerbating ischemia—particularly when proximal lesions are left untreated (28, 29).

Given these complexities, reliance solely on preserved FFR (>0.80) may underestimate true ischemic burden in MB-associated lesions. A more nuanced diagnostic approach is warranted—one that incorporates anatomical imaging with IVUS and phase-specific physiologic assessment using indices such as iFR or dFFR. This is especially important in patients with persistent symptoms or inconclusive clinical findings.

#### 4.1.3 Implications of PCI in FFR ≤ 0.80 group

In contrast, revascularization conferred clear clinical benefit in patients with functionally significant lesions, as indicated by an FFR ≤ 0.80. These patients experienced significantly lower rates of MACE and angina-related rehospitalizations. Multivariate analysis confirmed PCI as an independent protective factor, reinforcing its therapeutic value in addressing hemodynamically relevant proximal lesions—even when complicated by MB.

Our results further suggest that when the FFR falls below 0.80, the combined ischemic burden from proximal atherosclerotic narrowing and MB-induced compression becomes clinically significant and warrants intervention. In this context, DES implantation can restore antegrade flow, stabilize vulnerable plaque, and improve long-term outcomes. Crucially, these data also emphasize the importance of procedural precision in MB-associated lesions. While a reduced FFR supports revascularization, operators must account for the contractile behavior of MB segments. Careful delineation of stent landing zones is essential to avoid complications arising from stent placement within dynamically compressed arterial regions.

### 4.2 PCI in proximal LAD stenosis involving myocardial bridging

#### 4.2.1 Risks associated with stent extension into MB segment

Our analysis demonstrated that patients receiving stent implantation extending into the MB segment exhibited a significantly higher incidence of MACE, with recurrent angina being the most prominent contributor. This observation is consistent with earlier studies reporting the mechanical and biological challenges of deploying stents within segments subject to dynamic systolic compression (30–33). The systolic narrowing characteristic of MB generates oscillatory shear forces and repetitive endothelial injury, which are known to trigger neointimal proliferation and increase the risk of in-stent restenosis (32, 33). Additionally, sustained extrinsic compression may impair endothelial healing, stimulate smooth muscle cell migration and proliferation, and compromise long-term stent integrity (33). Notably, in our cohort, several patients with no angiographic evidence of restenosis continued to experience exertional angina during follow-up. This suggests that residual dynamic compression within the MB segment may persist even after technically successful stent deployment, leading to ongoing ischemic symptoms despite the absence of fixed luminal narrowing (33).

#### 4.2.2 Anatomical predictors of poor outcomes after MB stenting

Subgroup analysis identified two critical anatomical factors associated with adverse outcomes in patients who received stents extending into the MB segment. First, a shorter spatial interval between the proximal stenosis and the bridged segment significantly increased the risk of MACE. This is likely attributable to the technical challenge of achieving precise stent deployment without encroaching on the MB region (9). Prior studies have indicated that when this distance is less than 10 mm, the likelihood of unintended stent protrusion into the MB segment rises substantially, thereby elevating the risk of mechanical complications (34). Second, the unique anatomical environment of MB—where the coronary artery is enveloped by contracting myocardium—renders it particularly susceptible to poor outcomes if a dissection extends into this region. Ongoing systolic compression within the bridged segment can intensify local shear forces and impede the natural resolution of dissections, potentially resulting in stent malapposition, thrombotic events, or in-stent restenosis (27, 32, 33). These findings highlight the essential role of IVUS in procedural planning. Beyond detecting otherwise unrecognized dissections, IVUS is instrumental in accurately delineating optimal proximal and distal landing zones, thereby reducing the likelihood of stent-related complications and enhancing procedural safety (32, 33).

#### 4.2.3 Recommendations for stenting strategy in LAD-MB lesions

In light of the elevated risk of complications associated with stent implantation within MB segments, accumulating evidence favors a “no-stent zone” approach—deliberately avoiding stent deployment in dynamically compressed coronary segments (32, 33). Abdalwahab et al. proposed that extending the stent at least 3–5 mm beyond both the proximal and distal margins of the MB segment may reduce mechanical strain on the stent structure and enhance clinical outcomes (32). This technique minimizes the adverse effects of recurrent systolic compression, which can otherwise cause cyclic mechanical injury, impair endothelial healing, and promote neointimal hyperplasia—mechanisms central to restenosis and late stent failure (27, 33). In their study, the application of second-generation DES under IVUS guidance, combined with deliberate avoidance of the bridged segment, resulted in an absence of stent fractures or restenosis during long-term follow-up. In contrast, findings from our cohort revealed that direct stenting within the MB—without such anatomical consideration—was associated with significantly higher rates of MACE.

To improve procedural outcomes in this anatomically complex subset, a dual-modality strategy is advisable. Integration of IVUS for structural assessment with advanced physiologic evaluation—such as dFFR or dobutamine-stress FFR—facilitates both accurate lesion characterization and ischemia confirmation (9, 32). This comprehensive pre-intervention framework supports optimal landing zone selection and reduces the likelihood of inadvertently covering high-risk bridged segments. A systematic planning protocol that incorporates IVUS-defined MB morphology and dFFR-derived ischemic mapping may allow for tailored stent placement strategies, minimize procedural hazards, and align interventions with individual patient vascular physiology (9, 32).

### 4.3 Limitations

This study has several limitations. It was a retrospective, single-center study conducted in a Chinese population, which may introduce selection bias and significantly limit the generalizability of the findings to other ethnicities, regions, or healthcare systems. The sample size—especially in the MB-stent subgroup—was relatively small, potentially underpowering the detection of rare events. Although IVUS was routinely used, advanced functional indices such as such as dFFR, hyperemic iFR, or dobutamine stress testing were not consistently applied or available. This may have led to an underestimation of ischemia in borderline or discordant FFR cases, particularly in the context of MB, where dynamic systolic compression can impair diastolic flow that is not captured by conventional cycle-averaged FFR. This may have led to an underestimation of ischemia in borderline or discordant FFR cases, particularly in the context of MB, where dynamic systolic compression can impair diastolic flow that is not captured by conventional cycle-averaged FFR. Additionally, only mid-LAD myocardial bridges were evaluated, which may not reflect other anatomical variants such as proximal or distal MBs, further limiting the extrapolation of our findings to the broader MB population. Moreover, certain anatomical parameters such as MB depth were not routinely quantified from IVUS due to the absence of standardized definitions and technical challenges in reliable measurement. This limitation may have restricted our ability to assess additional structural predictors of clinical outcomes. Furthermore, no formal correction for multiple testing was applied in subgroup analyses, as only a single predefined stratification (stent extension into MB) was conducted. However, the results should be interpreted as exploratory and hypothesis-generating, and the potential risk of type I error is acknowledged. In addition, further subgroup analyses comparing deferred patients with and without stent extension into the MB were not performed due to sample size constraints and the exploratory nature of the study. Finally, the follow-up period, while adequate for intermediate outcomes, may not capture late complications such as very late stent thrombosis or vessel remodeling. A randomized controlled trial (although challenging in this clinical setting) would be useful to further validate these findings.

## 5 Conclusion

In patients with moderate MB and proximal LAD stenosis, an FFR-guided strategy combined with IVUS optimization was associated with improved clinical outcomes. PCI was beneficial when FFR ≤ 0.80, but stent extension into the MB segment led to significantly worse prognosis, particularly when anatomical risk factors were present. Interestingly, patients with FFR > 0.80 managed conservatively experienced a higher incidence of MACE, suggesting that conventional FFR may underestimate ischemia in the context of MB. This likely reflects the limitations of cycle-averaged FFR in detecting diastolic flow impairment caused by dynamic systolic compression. These results underscore the importance of integrating functional assessment with precise anatomical imaging to guide intervention. Avoiding stent coverage of dynamically compressed segments and tailoring landing zones with IVUS may reduce complications. A multimodal, physiology-anatomy–based approach is therefore essential in this complex lesion subset, where reliance on FFR alone may be insufficient.

## Data Availability

The original contributions presented in the study are included in the article/Supplementary Material, further inquiries can be directed to the corresponding authors.
